# APC selectively mediates response to chemotherapeutic agents in breast cancer

**DOI:** 10.1186/s12885-015-1456-x

**Published:** 2015-06-07

**Authors:** Monica K. VanKlompenberg, Claire O. Bedalov, Katia Fernandez Soto, Jenifer R. Prosperi

**Affiliations:** 1 Harper Cancer Research Institute, A134 Harper Hall, 1234 Notre Dame Ave., South Bend, IN 46617 USA; 2 Department of Biochemistry and Molecular Biology, Indiana University School of Medicine – South Bend, South Bend, IN USA; 3 Department of Biological Sciences, University of Notre Dame, Notre Dame, IN USA

**Keywords:** Adenomatous polyposis coli, Breast cancer, Chemotherapy, Src

## Abstract

**Background:**

The *Adenomatous Polyposis Coli* (*APC*) tumor suppressor is mutated or hypermethylated in up to 70 % of sporadic breast cancers depending on subtype; however, the effects of *APC* mutation on tumorigenic properties remain unexplored. Using the *Apc*^*Min/+*^ mouse crossed to the Polyoma middle T antigen (PyMT) transgenic model, we identified enhanced breast tumorigenesis and alterations in genes critical in therapeutic resistance independent of Wnt/β-catenin signaling. *Apc* mutation changed the tumor histopathology from solid to squamous adenocarcinomas, resembling the highly aggressive human metaplastic breast cancer. Mechanistic studies in tumor-derived cell lines demonstrated that focal adhesion kinase (FAK)/Src/JNK signaling regulated the enhanced proliferation downstream of *Apc* mutation. Despite this mechanistic information, the role of APC in mediating breast cancer chemotherapeutic resistance is currently unknown.

**Methods:**

We have examined the effect of *Apc* loss in MMTV-PyMT mouse breast cancer cells on gene expression changes of ATP-binding cassette transporters and immunofluorescence to determine proliferative and apoptotic response of cells to cisplatin, doxorubicin and paclitaxel. Furthermore we determined the added effect of Src or JNK inhibition by PP2 and SP600125, respectively, on chemotherapeutic response. We also used the Aldefluor assay to measure the population of tumor initiating cells. Lastly, we measured the apoptotic and proliferative response to *APC* knockdown in MDA-MB-157 human breast cancer cells after chemotherapeutic treatment.

**Results:**

Cells obtained from MMTV-PyMT;*Apc*^*Min/+*^ tumors express increased MDR1 (multidrug resistance protein 1), which is augmented by treatment with paclitaxel or doxorubicin. Furthermore MMTV-PyMT;*Apc*^*Min/+*^ cells are more resistant to cisplatin and doxorubicin-induced apoptosis, and show a larger population of ALDH positive cells. In the human metaplastic breast cancer cell line MDA-MB-157, *APC* knockdown led to paclitaxel and cisplatin resistance.

**Conclusions:**

APC loss-of-function significantly increases resistance to cisplatin-mediated apoptosis in both MDA-MB-157 and the PyMT derived cells. We also demonstrated that cisplatin in combination with PP2 or SP600125 could be clinically beneficial, as inhibition of Src or JNK in an *APC*-mutant breast cancer patient may alleviate the resistance induced by mutant *APC*.

**Electronic supplementary material:**

The online version of this article (doi:10.1186/s12885-015-1456-x) contains supplementary material, which is available to authorized users.

## Background

Breast cancer is the leading cause of cancer-related death in non-smoking women, and the most commonly diagnosed cancer in women in the United States [1]. Despite the tremendous amount of information about the etiology of breast cancer, many questions remain unanswered. Over the past 10–15 years, it has become evident that there are multiple different subtypes of breast cancer that respond differently to chemotherapeutic and targeted therapies [2]. Importantly, patient survival can be predicted by a variety of factors, including subtype and responsiveness to standard chemotherapeutic agents ([3–5] and reviewed in [6]).

The *Adenomatous Polyposis Coli* (by convention, the human and mouse genes are *APC* and *Apc*, respectively, whereas the protein from all species is APC) tumor suppressor is mutated or silenced by hypermethylation in up to 70 % of sporadic breast cancers depending on subtype ([7, 8] reviewed in [9]). APC loss-of-function most commonly leads to activation of the Wnt/β-catenin pathway. Multiple, less investigated roles of APC that occur independent of Wnt activation, such as regulation of proliferation, epithelial polarity, cytoskeletal organization, microtubule stability, and DNA repair (reviewed in [9]) suggest that APC may be important in therapeutic responsiveness. There are several proposed mechanisms by which chemotherapeutic resistance is conferred within cancer including both host factors and genetic or epigenetic alterations [10]. ATP-binding cassette transporters have drawn significant attention because of their ability to regulate drug accumulation within cells. In *Apc*-mutant mouse mammary glands during lactation, expression of the ATP-binding cassette sub-family G member 2/Breast cancer resistance protein (ABCG2/BCRP) was altered [11], suggesting that APC may regulate therapeutic resistance through mediation of ATP-binding cassette transporters. In addition, tumor initiating cells (TICs) have increased ATP-binding cassette pumps [12] and may also be an important part of chemotherapeutic resistance. Understanding how APC mediates sensitivity of breast tumor cells to chemotherapy is crucial to future breast cancer treatment, as the tumors that arise in *Apc-*mutant MMTV-PyMT mice resemble human metaplastic breast cancer, an aggressive subtype of triple-negative breast cancer (TNBC) with limited targeted therapies.

While breast cancer treatment varies by subtype, there are standard therapies that are currently employed depending upon subtype. The oncogenic events and signaling pathways that drive these tumor subtypes are distinct, indicating that a better understanding of their molecular basis will provide opportunities for predicting response to chemotherapy and the development of novel therapeutic approaches, to ultimately improve patient outcomes. Hormone responsive breast cancers are treated with specific anti-estrogens, such as tamoxifen, or monoclonal antibodies to HER2, such as trastuzumab. TNBC are treated with a combination of surgery, radiation, and systemic cytotoxic chemotherapeutic agents [13] with a combination of taxane and anthracycline drugs being used as the first line of defense [14]. In this study, we have explored three chemotherapeutic agents with different modes of action that are commonly used to treat TNBC. Despite the differing mechanisms, paclitaxel, doxorubicin, and cisplatin are used at various stages (neoadjuvant, adjuvant, metastatic) to treat breast cancer [15–19], [20, 21]. Furthermore, cisplatin treatment has been shown to mediate tumor response in the presence of mutant *APC* in other tumor types [22].

We previously demonstrated that *Apc* mutation accelerates the MMTV-PyMT model of breast tumorigenesis independent of Wnt/β-catenin signaling [23]. We made the novel observation that focal adhesion kinase (FAK)/Src/JNK signaling was enriched and required for the enhanced proliferation [23]. Herein we report that APC loss-of-function in cells from the MMTV-PyMT mouse model and metaplastic human breast cancer cell line MDA-MB-157 results in resistance to chemotherapy-induced apoptosis. *Apc* mutation in cells from the MMTV-PyMT mouse model also results in increased expression of MDR1 and a greater population of TICs.

## Methods

### Cell culture

MMTV-PyMT;*Apc*^*+/+*^ and MMTV-PyMT;*Apc*^*Min/+*^ cells were isolated as previously described [23] and were grown in RPMI 1640 media supplemented with 10 % fetal bovine serum, 1 % penicillin/streptomycin and 1:5000 plasmocin (Invivogen, San Diego, CA). MDA-MB-157 breast cancer cells (ATCC, Manassas, VA) were maintained in RPMI 1640 media supplemented with 10 % fetal bovine serum, 1 % penicillin/streptomycin, 25 mM HEPES and 1:5000 plasmocin. All cells were routinely passaged using 0.25 % trypsin/EDTA and maintained at 37 °C with 5 % CO_2_. MDA-MB-157 cells were subjected to lentiviral mediated shRNA knockdown of *APC* using two different MISSION shRNA *APC* constructs (Sigma-Aldrich, St Louis, MO). After transduction, cells were maintained in media containing 1.5 μg/mL puromycin (Sigma-Aldrich).

### Drug treatment

Cells were treated for 24 h with each chemotherapeutic agent or solvent control: doxorubicin (MP Biomedicals, LLC, Santa Ana, CA), paclitaxel (Sigma-Aldrich) or cisplatin (cis-Diammineplatinum (III) dichloride, Sigma-Aldrich). Drug concentrations for MMTV-PyMT-derived cells were 2.5 μM paclitaxel, 16 μM cisplatin, or 500 nM doxorubicin. MDA-MB-157 cells were treated with 0.078 μM paclitaxel, 4 μM cisplatin or 12.5 nM doxorubicin. These drug doses were selected after treatment of the MMTV-PyMT;*Apc*^*+/+*^ cells from 24–72 h showed approximately a 50 % reduction in cell population (data not shown). For the combination treatments, chemical inhibitors were added to the media 18 h after chemotherapeutic agents, resulting in a 6 h treatment with a combination of cisplatin or doxorubicin and 50 μM PP2 (Src inhibitor, Sigma-Aldrich) or 50 μM SP600125 (JNK inhibitor, Sigma-Aldrich). For BrdU incorporation assays, treatment was the same as above with the addition of 5-bromo-2’-deoxyuridine (BrdU, 10 μM, BD Pharmigen, Franklin Lakes, NJ) 8 h after chemotherapeutic agents.

### Immunofluorescence

For all experiments, cells were seeded in 12 well plates on glass coverslips for 24 h prior to treatment. For cell proliferation via BrdU incorporation, apoptosis via cleaved caspase 3 and APC immunofluorescence, cells were fixed in 3.7 % formaldehyde for 15 min and then permeabilized in 0.3 % Triton X-100 for 15 min. Immunofluorescence (IF) for BrdU was performed as previously described [23]. All antibodies were diluted in blocking buffer that consisted of 0.2 % non-fat dry milk, 2 % Bovine Serum Albumin and 0.3 % Triton X-100 in Phosphate Buffered Saline (PBS). Cells were incubated with primary antibodies: anti-BrdU rat monoclonal antibody (1:300, Abcam, Cambridge, MA), anti-cleaved caspase 3 rabbit monoclonal antibody (1:400, Cell Signaling Technology, Danvers, MA) or anti-APC (1:400, a gift from K. Neufeld, University of Kansas) for 1 h at 37 °C. Following washes in PBS, samples were incubated in the appropriate secondary antibody: rhodamine conjugated goat anti-rat (1:100, Thermo Scientific, Rockford, IL) or goat-anti-rabbit Alexa Fluor 488 (1:1000, Life Technologies, Carlsbad, CA). F-actin was visualized by co-staining with fluorescently conjugated Phalloidin (1:200, Life Technologies) and slides were mounted with Fluoromount G with Hoescht. The percent of positive cells was determined for each assay with at least 150 cells being counted per condition. Each assay was run in triplicate and repeated three times.

### RNA isolation and RT-PCR

MMTV-PyMT;*Apc*^*+/+*^ and MMTV-PyMT;*Apc*^*Min/+*^ cells were seeded at 2.4 x10^5^ cells/well in 6 well plates for 24 h then treated with drugs as above for 24 h. RNA was isolated using Tri Reagent (Molecular Research Center, Cincinnati, OH) and cDNA synthesis was performed with iScript from 1 μg RNA (Bio-Rad Laboratories, Hercules, CA). Real-time RT-PCR was performed using Power SYBR Green master mix (Applied Biosystems, Foster City, CA), 50 ng of cDNA, and 7.5 μM of each primer (primer sequences are presented in Table 1). The amplification program included 2 initial steps at 50 °C for 2 min and 95 °C for 10 min followed by 40 cycles of 95 °C for 15 s and 60 °C for 1 min followed by generation of a melt curve (CFX Connect 96 thermal cycler, Bio-Rad). Samples were run in duplicate and 18 s rRNA was used as a reference gene for all mouse studies. Microarray analysis on MDR1 and ABCG2 was previously described [11]. The knockdown of *APC* in the MDA-MB-157 cells was verified via quantitative real time PCR following the same protocol with GAPDH as the reference gene.

**Table 1:** Quantitative real time PCR primer sequences

Gene Name	Forward Primer (5’ – 3’)	Reverse Primer (5’ – 3’)
m18s	GGCGGCTTGGTGACTCTAGAT	CTTCCTTGGATGTGGTAGCCG
mMDR1	CATTGGTGTGGTGAGTCAGC	CTCTCTCTCCAACCAGGGTC
mABCG2	GCCAGTCTATGTTACCTCTTTCTGTCA	TGCATTCCAGCGGCATCATATTTCA
hGAPDH	GAAGGTGAAGGTCGGAGTC	GAAGATGGTGATGGGATTTC
hAPC	TGTCCCGTTCTTATGGAA	TCTTGGAAATGAACCCATAGG

Species is indicated by either an m (mouse) or h (human) before the gene name

### Western blots

Total protein was isolated from MMTV-PyMT tumor-derived cells after 24 h treatment with the same chemotherapeutic agents as above using lysis buffer (20 mM Tris–HCl, 150 mM NaCl, 1 % Triton-X, 0.5 % NP-40, 50, mM NaF, 1 mM Na_3_VO_4_, 5 mM Sodium pyrophosphate, 0.2 mM PMSF, 1x protease inhibitor cocktail (Fisher) and 1x phosphatase inhibitor cocktail 2 (Sigma). 30 μg of protein were separated by SDS-PAGE (8 % gel), and transferred onto Immobilon-P membrane (Millipore). After transfer, membranes were blocked in 5 % non-fat dry milk in 1 % TBS with 0.1 % Tween (TBS-T) for 1 h at room temperature. Blots were incubated with the primary antibody (MDR1, 1:1000 in 1 % bovine serum albumin in TBS-T, Cell Signaling, or ABCG2, 1:100 in 5 % non-fat dry milk in TBS-T, Abcam) for 2 nights at 4 °C or β-actin (1:25000, 1 % BSA in TBS-T, Sigma) for 1 h at room temperature. Secondary antibody (anti-rabbit, anti-rat or anti-mouse IgG-HRP, 1:1000) was diluted in the same diluent as the corresponding primary antibody and incubated at room temperature for 1 h. Blots were developed with Clarity ECL regent (Bio-rad) and a ChemiDoc MP Imaging System (Bio-rad). Densitometry quantification was performed using ImageJ software (NIH). Blots are representative of three runs.

### ALDEFLUOR assays

Aldehyde dehydrogenase (ALDH) enzyme activity was determined using the ALDEFLUOR™ Kit (Stem Cell Technologies, Vancouver, British Columbia, Canada) according to the manufacturer’s protocol. Briefly 2.0x10^5^ MMTV-PyMT;*Apc*^*+/+*^ or MMTV-PyMT;*Apc*^*Min/+*^ cells were suspended in Aldefluor™ assay buffer containing ALDH substrate (Bodipy-Aminoacetaldehyde) which served as the “test” sample. An identical sample served as the “control” containing the ALDH substrate and diethylaminobenzaldehyde (DEAB), a specific ALDH1 enzyme inhibitor. Both the test and control samples were incubated for 60 min at 37 °C. The fluorescent ALDH-expressing cells were detected in the green fluorescence channel (515–535 nm) of a Cytotomics FC 500 (Beckman Coulter, Brea, CA) flow cytometer. Data were analyzed using FlowJo Flow Cytometry Data Analysis Software (Tree Star, Ashland, OR). The percent shift between gated events in the test versus control samples was calculated; a greater shift indicates a greater number of ALDH positive cells in a given sample.

### Statistical analysis

Student’s t-tests were used for all analyses except the combination treatments where a two-way ANOVA with Bonferroni post-hoc test was used. A p-value < 0.05 was considered significant.

## Results

**Fig. 1:**
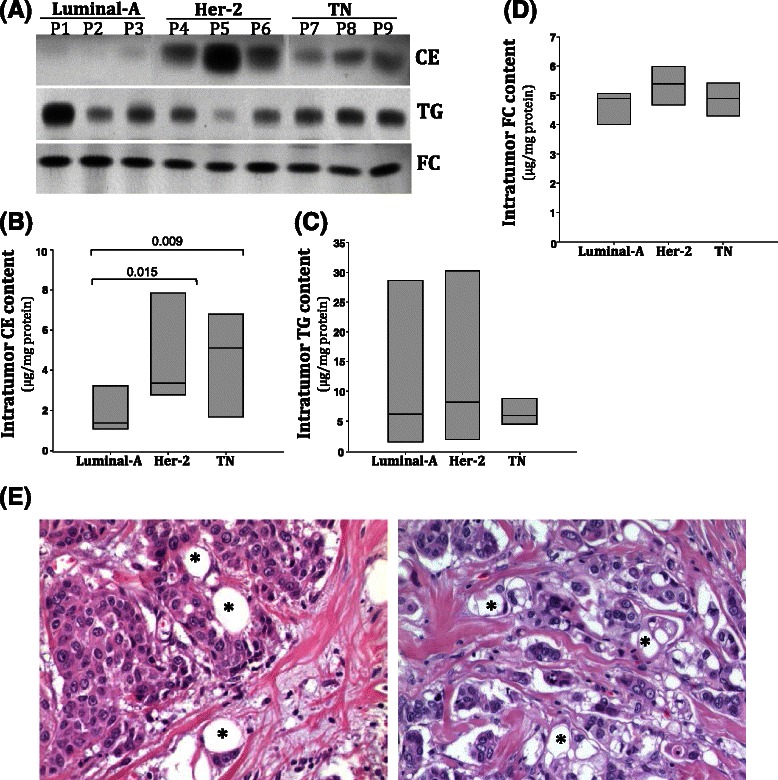
Gene expression of ATP-dependent binding cassette transporters. **a** Microarray analysis of mammary glands from *Apc*^*Min/+*^ and *Apc*^*+/****+***^ mice at d16 of lactation show a decrease in ABCG2 and increase in MDR1 expression due to *Apc* mutation. **b** MDR1 gene expression in cells from MMTV-PyMT;*Apc*^*Min/+*^ and MMTV-PyMT;*Apc*^*+/****+***^ mice after 24 h treatment with either solvent control, paclitaxel, cisplatin or doxorubicin. MDR1 expression was significantly increased in cells from MMTV-PyMT;*Apc*^*Min/+*^ mice after treatment with paclitaxel and doxorubicin but not cisplatin. **c** ABCG2 gene expression in cells from MMTV-PyMT;*Apc*^*Min/+*^ and MMTV-PyMT;*Apc*^*+/****+***^ mice after treatment for 24 h with either solvent control, paclitaxel, cisplatin or doxorubicin. ABCG2 expression was not different between MMTV-PyMT;*Apc*^*Min/+*^ and MMTV-PyMT;*Apc*^*+/+*^ cells and chemotherapy treatment had no effect on ABCG2 expression. **d** Representative western blots for MDR1 and ABCG2 in cells from MMTV-PyMT;*Apc*^*Min/+*^ and MMTV-PyMT;*Apc*^*+/****+***^ mice after treatment for 24 h with either solvent control, paclitaxel, cisplatin or doxorubicin. **e** Quantification of MDR1 western blots shows that MMTV-PyMT;*Apc*^*Min/+*^ cells have enhanced MDR1 expression when treated with doxorubicin. **f** Quantification of ABCG2 western blots shows that MMTV-PyMT;*Apc*^*+/+*^ cells have elevated ABCG2 protein expression compared to MMTV-PyMT;*Apc*^*Min/+*^ cells. Results in B, C, E and F are shown as the means ± SEM from 3 independent experiments; *P < 0.05 when comparing MMTV-PyMT;*Apc*^*Min/+*^ to MMTV-PyMT;*Apc*^*+/+*^ cells and #P < 0.05 when comparing MMTV-PyMT;*Apc*^*Min/+*^ cells treated with solvent control or chemotherapy agent.

One major mechanism of chemotherapeutic resistance is the presence of ATP-dependent efflux pumps to regulate the rate at which chemotherapeutic drugs are effluxed out of a cell and control the amount of drug remaining within the cell [10]. Through microarray analysis, our laboratory previously demonstrated that ABCG2 expression was down-regulated in *Apc-*mutant mouse mammary glands during lactation [11]; however, MDR1 expression was significantly increased (Fig. 1a). MDR1 is one of the most common efflux pumps that confers chemotherapeutic resistance and has been shown to regulate efflux of paclitaxel and doxorubicin [12]. Consistent with the developmental studies (Fig. 1a), cells isolated from MMTV-PyMT;*Apc*^*Min/+*^ tumors showed a significant increase of MDR1 expression compared to control MMTV-PyMT;*Apc*^*+/+*^ cells (Fig. 1b, Additional file 1). Treatment with paclitaxel and doxorubicin, but not cisplatin, further enhanced expression of MDR1 in MMTV-PyMT;*Apc*^*Min/+*^ cells (Fig. 1b, Additional file 1). Doxorubicin treatment also enhanced MDR1 protein expression in MMTV-PyMT;*Apc*^*Min/+*^ cells (Fig. 1d and e, Additional file 1). We observed no changes in the expression of ABCG2 after treatment with chemotherapeutic agents (Fig. 1c, Additional file 1). However MMTV-PyMT;*Apc*^*+/+*^ have a greater expression of ABCG2 protein compared to MMTV-PyMT;*Apc*^*Min/+*^ cells (Fig. 1d and f, Additional file 1).

**Fig. 2:**
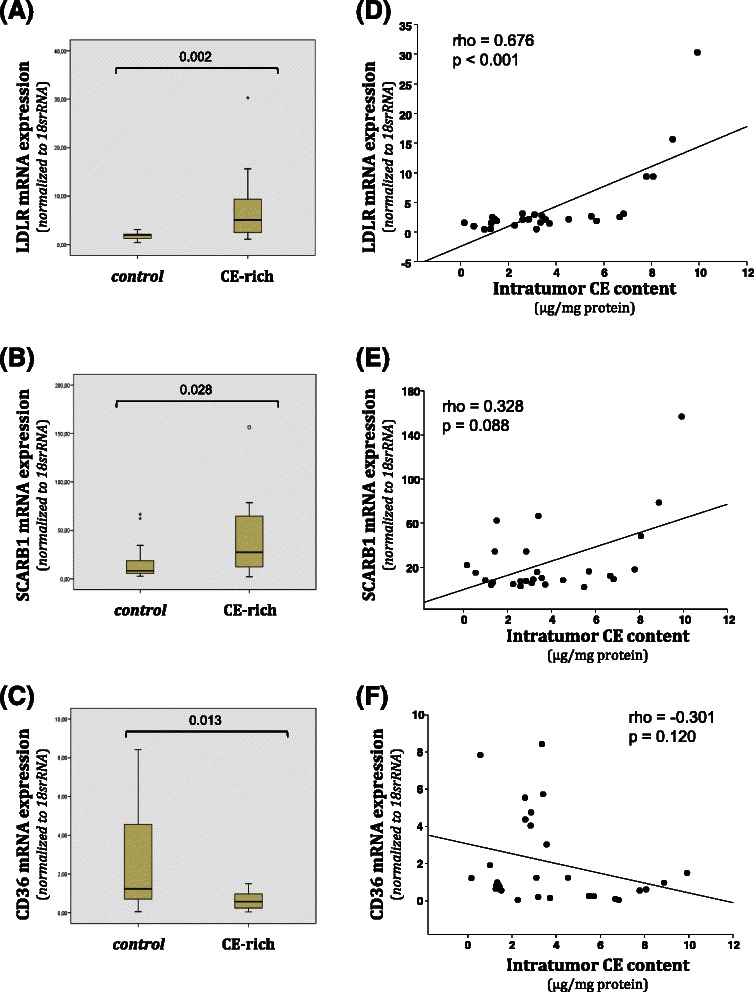
Cell proliferation and apoptosis in MMTV-PyMT;*Apc*^*Min/+*^ and MMTV-PyMT;*Apc*^*+/+*^ cells after treatment with paclitaxel, cisplatin and doxorubicin. **a** Cell proliferation as measured by BrdU incorporation after chemotherapuetic treatment. MMTV-PyMT;*Apc*^*Min/+*^ cells showed a modest decrease in proliferation after treatment with cisplatin and doxorubicin compared to MMTV-PyMT;*Apc*^*+/+*^ cells. **b** Apoptosis as measured by cleaved caspase 3 immunofluorescence (IF). The percentage of apoptosis was lower in cisplatin and doxorubicin treated MMTV-PyMT;*Apc*^*Min/+*^ compared to MMTV-PyMT;*Apc*^*+/+*^ cells while paclitaxel treatment did not affect apoptosis levels. **c** Representative images of cleaved caspase 3 (CC3) IF. The scale bar is equal to 200 microns. White arrows are representative cleaved caspase 3 positive cells. Data are shown as the means ± SEM from 3 independent experiments; *P < 0.05

Given the increase in MDR1 expression, we hypothesized that *Apc* mutation may therefore confer chemotherapeutic resistance. To test this hypothesis, we measured cell proliferation and apoptosis after a 24-hour treatment with cisplatin, paclitaxel or doxorubicin. BrdU incorporation demonstrated that while paclitaxel treatment did not alter cell proliferation, the impact of cisplatin and doxorubicin on proliferation was modestly enhanced in MMTV-PyMT;*Apc*^*Min/+*^ cells (Fig. 2a, Additional file 1). Given the mechanisms of action of chemotherapeutic agents, and the lack of a strong proliferative effect, we used cleaved caspase 3 IF to determine whether *Apc* mutation decreased the rate of apoptosis after chemotherapeutic treatment. As expected, no difference was observed in the percent of apoptosis in control (vehicle-treated) MMTV-PyMT;*Apc*^*Min/+*^ versus MMTV-PyMT;*Apc*^*+/+*^ cells (Fig. 2b and c, Additional file 1). However, MMTV-PyMT;*Apc*^*Min/+*^ cells had decreased cisplatin- or doxorubicin-induced apoptosis compared to MMTV-PyMT;*Apc*^*+/+*^ (Fig. 2b and c, Additional file 1). No difference in paclitaxel-induced apoptosis was observed in the two cell lines.

**Fig. 3:**
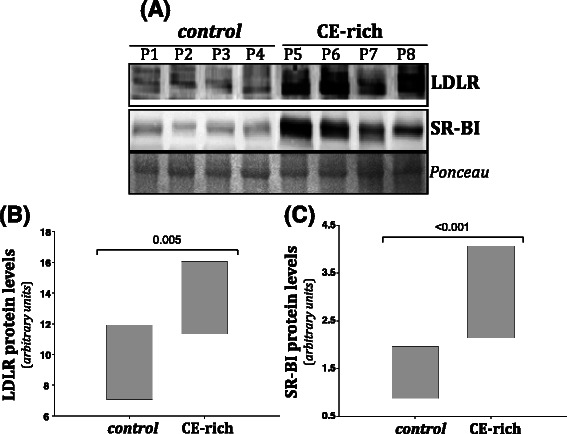
Apoptosis in MMTV-PyMT;*Apc*^*Min/+*^ and MMTV-PyMT;*Apc*^*+/+*^ cells treated with chemotherapeutic drugs and targeted inhibitors. **a** Apoptosis was measured by cleaved caspase 3 IF in the presence of cisplatin. Treatment with cisplatin and either PP2 or SP600125 significantly increases apoptosis compared to cisplatin alone in MMTV-PyMT;*Apc*^*Min/+*^cells. No effect was observed with the addition of PP2 or SP600125 in the MMTV-PyMT;*Apc*^*+/+*^cells. **b** Apoptosis was measured by cleaved caspase 3 IF with doxorubicin treatments. **c** Representative cleaved caspase 3 IF images of cells treated with cisplatin and the targeted inhibitor. The scale bar is equal to 200 microns and arrows are used to depict specific cleaved caspase 3 (CC 3) positive cells in each image. Data are shown as the means ± SEM from 3 independent experiments; *P < 0.05 when comparing MMTV-PyMT;*Apc*^*Min/+*^ to MMTV-PyMT;*Apc*^*+/+*^ cells and ** P < 0.05 when comparing the combination treatment versus a single agent (cisplatin or doxorubicin)

We previously demonstrated that mutant *Apc* in the MMTV-PyMT mouse model increased proliferation and expression of pFAK, pSrc and pJNK, and that inhibition of Src or JNK diminishes the APC-mediated cell proliferation [23]. Given the effect of *Apc* status on cisplatin- and doxorubicin-mediated apoptosis (Fig. 2b, Additional file 1) and to understand whether the Src/JNK signaling pathway could impact chemotherapeutic resistance, we measured apoptosis in cells treated with a combination regimen. Cells were treated with either cisplatin or doxorubicin in combination with either an inhibitor to Src (PP2) or JNK (SP600125). The addition of PP2 or SP600125 to cisplatin significantly increased apoptosis compared to cisplatin alone in MMTV-PyMT;*Apc*^*Min/+*^cells (Fig. 3a and c, Additional file 1). Therefore the cisplatin-mediated apoptosis becomes equivalent in the two cell lines with the inhibition or Src or JNK. It is also evident that cisplatin and doxorubicin have differing modes of action because the doxorubicin-mediated apoptosis in the MMTV-PyMT;*Apc*^*Min/+*^ cells was not affected by the addition of PP2 or SP600125 (Fig. 3b, Additional file 1).

**Fig. 4:**
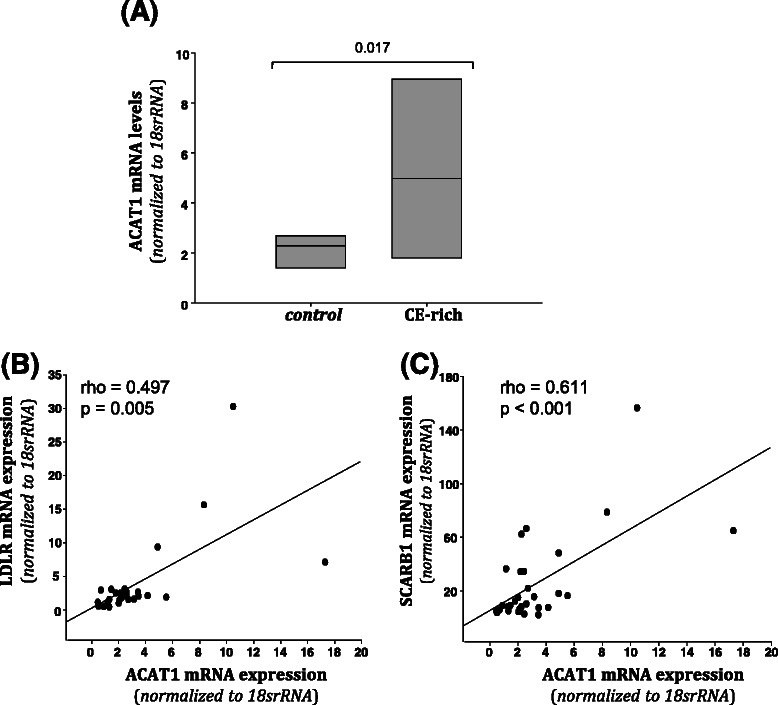
MMTV-PyMT;*Apc*^*Min/+*^ cells have higher aldehyde dehydrogenase (ALDH) enzyme activity than MMTV-PyMT;*Apc*^*+/+*^ cells. ALDH activity was measured using an Aldefluor™ Kit. For each cell line a Control (+DEAB) and test (−DEAB) sample were run. **a** Representative FACS analysis of ALDH activity in MMTV-PyMT;*Apc*^*+/+*^ and MMTV-PyMT;*Apc*^*Min/+*^ cells using the Aldefluor™ assay. ALDH activity is increased in MMTV-PyMT;*Apc*^*Min/+*^ cells. **b** The population of cells that shifted outside of the control population was calculated for each test sample, indicating ALDH activity. MMTV-PyMT;*Apc*^*Min/+*^cells show a larger percentage of cells shifted outside of the control range than MMTV-PyMT;*Apc*^*+/+*^cells. Data are shown as the means ± SEM from 3 independent experiments; *P < 0.05

TICs have been shown to have higher levels of ABC transporters or efflux pumps compared to normal differentiated cells [12], and impact chemotherapeutic resistance. In other words, the cells that often remain after treatment, and therefore cause tumor recurrence are often TICs. A common mechanism to measure TICs is through the use of the ALDEFLUOR assay using FACS, where 23 out of 33 human breast cancer cell lines were found to be positive [24]. Therefore we used the ALDEFLUOR assay to determine the population of TICs. Cells were incubated with ALDH substrate in the presence (control) or absence (test) of the ALDH enzyme inhibitor, diethylaminobenzaldehyde (DEAB). The gated (ALDH^−^) population in the control condition was unchanged in the *Apc*-mutant versus wild-type cells (Fig. 4a, 97.7 % compared to 95.5 %). However, in the absence of the ALDH inhibitor, the gated population changed from 77.8 % in the MMTV-PyMT;*Apc*^*+/+*^cells to a mere 20.3 % in the MMTV-PyMT;*Apc*^*Min/+*^ cells, indicating that *Apc* mutation increases the TIC population (Fig. 4) a and b.

**Fig. 5:**
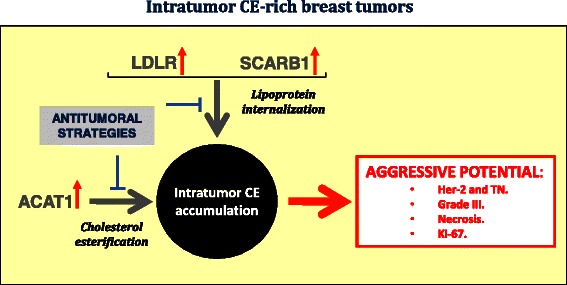
APC knockdown in MDA-MB-157 cells impacts response to paclitaxel and cisplatin. **a** Quantitative RT-PCR in MDA-MB-157 cells and shAPC constructs shows decreased level of *APC* in cells infected with the shAPC constructs. **b** Representative APC immunofluorescence images showing that APC knockdown cells have less APC protein compared to the MDA-MB-157 parent line. **c** Cell proliferation as measured by BrdU incorporation did not differ between the three cell lines after treatment with cisplatin, doxorubicin or paclitaxel. **d** Apoptosis as measured by cleaved caspase 3 IF. The percentage of apoptosis was lower in paclitaxel treated shAPC 1 and cisplatin treated shAPC 2 cells compared to MDA-MB-157 control cells. Doxorubicin treatment had no effect on rates of apoptosis. **e** Representative images of CC3 IF. Although there are a similar number of positive cells in many of the images, there are fewer total cells in those images representing treatments with a higher percent of apoptosis. The scale bar is equal to 100 microns (E) and 20 microns (B). Data are shown as the means ± SEM from 3 independent experiments; *P < 0.05

To translate our results from mouse cell lines to a human cell line, we turned to MDA-MB-157 cells, which are a triple negative breast cancer cell line derived from a pleural effusion from a metaplastic human breast cancer [25]. This specific cell line was chosen because of the metaplastic-like tumors that developed in the MMTV-PyMT;*Apc*^*Min/+*^ model [23]. As MDA-MB-157 cells have wild-type *APC* [26–27] we used lentiviral mediated shRNA to knockdown *APC* and observed a 60 % decrease in *APC* gene expression (Fig. 5a). Using immunofluorescence we observed that *APC* knockdown cells had decreased APC protein expression at the membrane and nucleus (Fig. 5b). Baseline cell proliferation was not changed in the *APC* knockdown cells (data not shown). Treatment with cisplatin, doxorubicin or paclitaxel did not impact proliferation in the *APC* knockdown lines compared to the parent line (Fig. 5c, Additional file 1), similar to the effects in the PyMT-derived model (Fig. 2). *APC* knockdown MDA-MB-157 cells exhibited decreased apoptosis after treatment with paclitaxel or cisplatin (Fig. 5d and e, Additional file 1). While paclitaxel response was not previously shown to be dependent on APC status (Fig. 2b), the impact of APC on cisplatin response (Fig. 2b, and 5d) is consistent between the two model systems. In contrast to the results in the PyMT model system (Figs. 2b) doxorubicin-mediated apoptosis was not altered by *APC*-status in the MDA-MB-157 cell system (Fig. 5d).

## Discussion

The recurrence of breast cancer, which accounts for 90 % of breast cancer-related deaths [13], is often accompanied by chemotherapeutic resistance [28]. Therefore, it is critical to investigate the biological markers involved with this process. Many mechanisms have been attributed to the development of chemotherapeutic resistance; however, one of the most well-known is the multidrug resistance theory. The expression of efflux pumps that regulate the rate in which drugs can remain in cells and increased expression or activity of these pumps leads to increased chemotherapeutic resistance. TICs or cancer stem cells express high levels of ABC transporters [12, 29] and are more resistant to chemotherapeutic agents than other tumor cells [30]. Our results in the mouse cell model support this claim because MMTV-PyMT;*Apc*^*Min/+*^ cells have increased TICs and higher levels of MDR1 expression. We previously reported that mutant *Apc* in the MMTV-PyMT model increased FAK activation [23]. FAK deletion has been shown to decrease the number of TICs present in both breast cancer [31–33] and skin cancer [34]. Therefore, the regulation of FAK by APC may be contributing to the change in the number of TICs through blocking the process of self-renewal.

ALDH-positive cells are more metastatic than ALDH-negative cells [24] and increased ALDH activity leads to poor clinical prognosis [35]. Cisplatin and paclitaxel resistant cell lines have increased ALDH expression compared to parent cells [36, 37]. ALDH positive cells have increased expression of STAT3 (signal transducer and activator of transcription 3) [38] which makes STAT3 an ideal target for decreasing the TIC population in MMTV-PyMT;*Apc*^*Min/+*^ cells. In addition to commercially available STAT3 inhibitors [39], miR-337-3p, a mature sequence of miR-337, can also inhibit STAT3, sensitizing lung cancer cells to paclitaxel treatment [40]. miR-337 is commonly overexpression in HER2-positive breast cancers [41] and non-inflammatory breast cancer samples from Tunisian women [42]. Another important factor is miR-135a, which binds to the *APC* gene [43, 44], and inhibits STAT3-induced pro survival gene expression and induces apoptosis in gastric cancer and lymphoma [45]. Future work will determine the impact of treatment with FAK and STAT3 inhibitors on the TIC population in MMTV-PyMT;*Apc*^*Min/+*^ cells as a means to regulate chemotherapeutic resistance.

The MMTV-PyMT;*Apc*^*Min/+*^ cells exhibited increased MDR1 expression, which would be expected to increase chemotherapeutic resistance. MDR1 expression in MMTV-PyMT;*Apc*^*Min/+*^ cells was augmented by doxorubicin and paclitaxel but not with cisplatin (Fig. 1), which is supported by currently published data that MDR1 effluxes both doxorubicin and paclitaxel but not cisplatin [12]. While studies have identified APC as a driver of multidrug resistance through the Wnt/β-catenin signaling in colorectal cancer [46–49], we have not thoroughly discussed this body of work since we previously reported the effects of *Apc* mutation in the MMTV-PyMT mouse model to be independent of Wnt/β-catenin signaling [23]. Microarray analysis of the NCI60 cell lines showed that there is an inverse relationship between drug resistance and MDR1 expression for 18 different compounds including doxorubicin and paclitaxel [50]. Doxorubicin-resistant MDA-MB-435 or MCF-7 cells [51–53] have increased MDR1, suggesting elevated MDR1 may be the mechanism by which *APC* mutation causes doxorubicin-resistance. Increased MDR1 will enhance doxorubicin efflux out of cells and thus decrease the intracellular drug accumulation. In addition, doxorubicin has been shown to increase MDR1 in B-cell lymphoma cells through activation of the Mitogen Activated Protein Kinase/Extracellular-signal-Regulated Kinases (MAPK/ERK) signaling pathway [54]. Conversely antibody-based microarray analysis determined that doxorubicin-resistant MDA-MB-231 have decreased protein expression of p-ERK1, cyclin B1 and cyclin D2 [55]. To our knowledge this is the first account of APC regulating basal and drug induced MDR1 expression in breast cancer. To link MDR1 expression to chemotherapeutic responsiveness and decreased apoptosis, future work will block expression of MDR1 using PSC833 [56], which has been previously used in breast cancer to inhibit MDR1 expression (reviewed by [57]).

Further studies are needed to delineate the mechanism responsible for the differences seen between doxorubicin and cisplatin, and the differences between the PyMT-derived cells and the MDA-MB-157 human breast cancer cell line. DNA damage repair mechanisms may also be affected by *Apc* mutation as cisplatin directly and doxorubicin indirectly cause DNA damage [58]. Mutation in *Apc* is known to increase a cell’s ability to repair mutated DNA [59, 60], and its interaction with DNA polymerase β (Pol-β) and flap endonuclease 1 (Fen-1) inhibits single nucleotide and long-patch excision repair [59, 61, 62]. Therefore we may expect that MMTV-PyMT;*Apc*^*+/+*^ cells might undergo apoptosis while MMTV-PyMT;*Apc*^*Min/+*^ cells would survive and these effects may vary by the direct (cisplatin) versus indirect (doxorubicin) effects on DNA damage.

Given that both models tested show that APC mediates response to cisplatin, future studies will elaborate on cisplatin resistance. Cisplatin resistance can include multiple pathways: increased drug efflux, evasion of apoptotic pathways, bypass of the replication checkpoint, increased cell proliferation and increased DNA damage repair [63], and thus it might be harder to delineate a specific mechanism. However, previous studies have demonstrated that APC mediates sensitivity to cisplatin in multiple tumor types including breast cancer [22]. We propose that one potential target may be drug efflux by, and expression of, ATP-binding cassette sub-family C member 2 (ABCC2) because it effluxes cisplatin [12] and can be found in breast cancer cells [12, 64]. This mechanism will need to be determined as we observed the most promising effect with combination treatment of cisplatin with Src or JNK inhibition. This is not the first report of drug resistance occurring via different mechanisms. In doxorubicin- and mitoxantrone- resistant MCF-7 breast cancer cells, both drugs led to decreased drug accumulation and enhanced drug efflux but only doxorubicin resistant cells had an increase in permeability factor glycoprotein-p170 (Pgp) expression [65]. Through microarray analysis both MCF-7 breast cancer cells and two different leukemia cell lines not only have increased expression of the ABC transporter reported to be overexpressed for each in the literature but also in several other efflux pumps [66]. This finding implies that it may be important to study multiple transporters in the case of chemotherapeutic resistance. There is redundancy in expression of different ABC transporters within resistant cells that may increase the difficulty in delineating which ABC transporters are explicitly driving chemotherapeutic resistance [50, 58].

The cisplatin-mediated changes in combination with PP2 and SP600125 corroborate results by others that demonstrate a synergistic effect between Src inhibition and cisplatin in breast and non-small lung cancer cell lines [67, 68]. The importance of Src in chemotherapeutic resistance was also demonstrated in human gallbladder adenocarcinoma cells where Src increased chemotherapeutic resistance by increasing the repair of cisplatin-DNA interstrand cross-links [69]. In addition to our data showing an enhanced response of *Apc-*mutant breast cancer cells to SP600125, previous studies have indicated a role of APC in mediating sensitivity to the JNK inhibitor VIII [22]. JNK has been shown to be important in cisplatin-mediated apoptosis in cell culture models for sarcoma [70] and lung cancer [71–73]. Moreover we saw similar cisplatin resistance in our human breast cancer cell line model, suggesting a global mechanism of resistance. These data imply that treatment of a patient with an *APC*-mutant breast cancer with Src or JNK inhibitors in combination with cisplatin would result in restored sensitivity to cisplatin.

Paclitaxel affects microtubule binding ([74, 75] and reviewed in [9]) and APC is required for the ability of EB1 to promote microtubule polymerization [76] by cooperating with EB1 to cap the plus ends of microtubules, thus preventing exchange of tubulin subunits [77]. APC deficiency in models of intestinal cancer strongly suppresses tumorigenesis due to a defect in microtubule stabilization [78]. Although *Apc* mutation in the PyMT-model did not result in paclitaxel resistance, we observed changes in MDR1 expression after paclitaxel treatment. Consistent with our data in MMTV-PyMT;*Apc*^*Min/+*^cells (Fig. 1), paclitaxel treatment upregulates MDR1 expression in MCF-7 breast cancer cells [79]. The pathway responsible for the paclitaxel-mediated MDR1 overexpression in MCF-7 breast cancer cells is via early growth response gene-1 (egr-1) [79]. While MDR1 expression was increased in the presence of paclitaxel treatment, no change in apoptosis was observed in *Apc*-mutant cells. Despite this, we have shown here that *APC* status in the MDA-MB-157 cells dictates resistance to paclitaxel. One potential reason for this discrepancy in the two cell lines may be related to the finding that in some cases the effects of paclitaxel has been linked to changes in autophagy and not apoptosis [80]. Inherent differences in gene expression profiles in the two model systems tested may also be responsible for the differences in chemo-responsiveness, and will be further investigated in the laboratory. Recently work has also shown that miR-135a contributes to paclitaxel resistance *in vitro* in cell culture models for non-small cell lung cancer, breast cancer and ovarian cancer while *in vivo* work with a non-small cell lung cancer model demonstrated a similar response [44]. This work is important to our study because miR-135a downregulates *APC* gene expression [43, 44] and thus may be responsible affecting chemotherapeutic resistance in our model systems.

## Conclusions

In this body of work we have shown that *Apc* mutation in the MMTV-PyMT mouse model leads to increased expression of MDR1, which is enhanced after treatment with doxorubicin and paclitaxel. *Apc* mutation also decreases cisplatin- or doxorubicin-mediated apoptosis. Treatment of *Apc-*mutant cells with a combination of cisplatin and Src or JNK inhibitors restores apoptosis to a level similar to the control cells. This is a significant finding that has the potential to eliminate chemotherapeutic resistance in *APC-*mutant breast cancers. Results from the human metaplastic breast cancer cell line MDA-MB-157 showed *APC* knockdown resulted in resistance to apoptosis in both cisplatin and paclitaxel. Taken together these results demonstrate that APC loss-of-function significantly impacts chemotherapeutic resistance.

## Additional file

Additional file 1: **This file contains tables for each quantitative graph in the paper.** Each table includes average, SEM and p-values for each cell line and treatment combination.

## Abbreviations

ABCG2/BCRP: ATP-binding cassette sub-family G member 2/Breast cancer resistance protein
ALDH: Aldehyde dehydrogenase
APC: Adenomatous polyposis coli
BrdU: 5-bromo-2’-deoxyuridine
CC3: Cleaved caspase 3
FAK: Focal adhesion kinase
JNK: c-Jun NH(2)-terminal kinase
IF: Immunofluorescence
MDR1: Multidrug resistance protein 1
MMTV-PyMT: Mouse mammary tumor virus-Polyoma middle T antigen
STAT3: Signal transduction and activator of transcription 3
Pgp: Permeability factor glycoprotein-p170
TIC: Tumor initiating cell
TNBC: Triple negative breast cancer
