# Erratum to: APC selectively mediates response to chemotherapeutic agents in breast cancer

**DOI:** 10.1186/s12885-016-2950-5

**Published:** 2016-11-28

**Authors:** Monica K. VanKlompenberg, Claire O. Bedalov, Katia Fernandez Soto, Jenifer R. Prosperi

**Affiliations:** 1 Harper Cancer Research Institute, A134 Harper Hall, 1234 Notre Dame Ave., South Bend, IN 46617 USA; 2 Department of Biochemistry and Molecular Biology, Indiana University School of Medicine – South Bend, South Bend, IN USA; 3 Department of Biological Sciences, University of Notre Dame, Notre Dame, IN USA

## Erratum

After publication of the original article [1], it is noticed that all the Figures in the HTML version of the article are incorrect, please see the correct figures below. We apologize for any inconvenience caused.

**Fig. 1:**
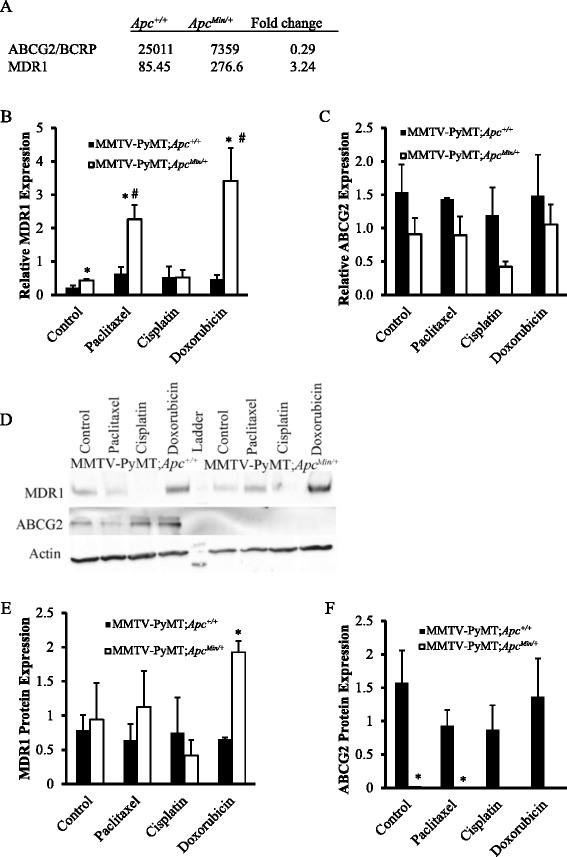
Gene expression of ATP-dependent binding cassette transporters. **a** Microarray analysis of mammary glands from *Apc* ^Min/+^ and *Apc* ^+/+^ mice at d16 of lactation show a decrease in ABCG2 and increase in MDR1 expression due to *Apc* mutation. **b** MDR1 gene expression in cells from MMTV-PyMT;*Apc* ^Min/+^ and MMTV-PyMT;*Apc* ^+/+^ mice after 24 h treatment with either solvent control, paclitaxel, cisplatin or doxorubicin. MDR1 expression was significantly increased in cells from MMTV-PyMT;*Apc* ^Min/+^ mice after treatment with paclitaxel and doxorubicin but not cisplatin. **c** ABCG2 gene expression in cells from MMTV-PyMT;*Apc* ^Min/+^ and MMTV-PyMT;*Apc* ^+/+^ mice after treatment for 24 h with either solvent control, paclitaxel, cisplatin or doxorubicin. ABCG2 expression was not different between MMTV-PyMT;*Apc* ^Min/+^ and MMTV-PyMT;*Apc* ^+/+^ cells and chemotherapy treatment had no effect on ABCG2 expression. **d** Representative western blots for MDR1 and ABCG2 in cells from MMTV-PyMT;*Apc* ^Min/+^ and MMTV-PyMT;*Apc* ^+/+^ mice after treatment for 24 h with either solvent control, paclitaxel, cisplatin or doxorubicin. **e** Quantification of MDR1 western blots shows that MMTV-PyMT;*Apc* ^Min/+^ cells have enhanced MDR1 expression when treated with doxorubicin. **f** Quantification of ABCG2 western blots shows that MMTV-PyMT;*Apc* ^+/+^ cells have elevated ABCG2 protein expression compared to MMTV-PyMT;*Apc* ^Min/+^ cells. Results in **b**, **c**, **e** and **f** are shown as the means ± SEM from 3 independent experiments; *P < 0.05 when comparing MMTV-PyMT;*Apc* ^Min/+^ to MMTV-PyMT;*Apc* ^+/+^ cells and #*P* < 0.05 when comparing MMTV-PyMT;*Apc* ^Min/+^ cells treated with solvent control or chemotherapy agent

**Fig. 2:**
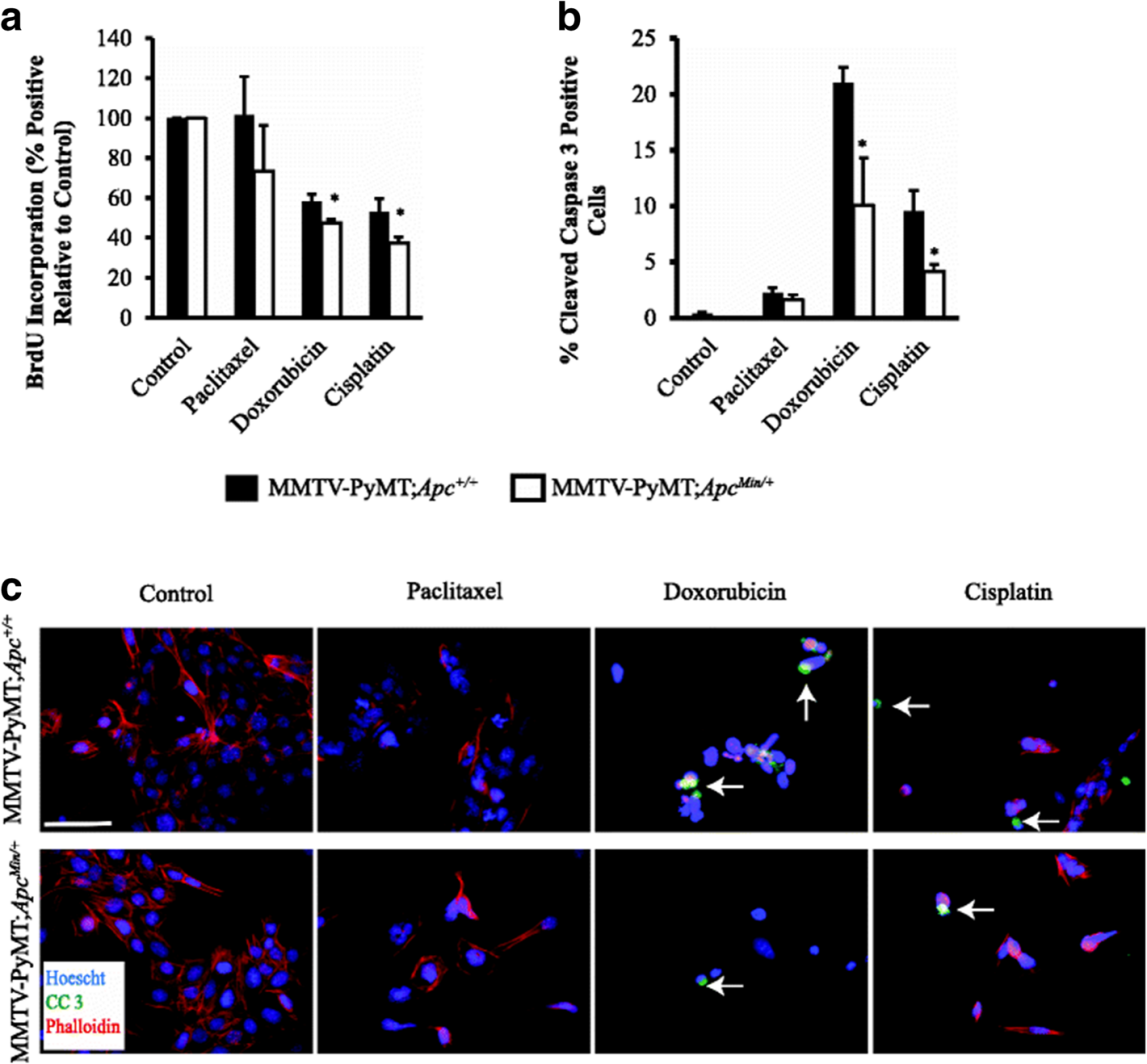
Cell proliferation and apoptosis in MMTV-PyMT;*Apc* ^Min/+^ and MMTV-PyMT;*Apc* ^+/+^ cells after treatment with paclitaxel, cisplatin and doxorubicin. **a** Cell proliferation as measured by BrdU incorporation after chemotherapuetic treatment. MMTV-PyMT;*Apc* ^Min/+^ cells showed a modest decrease in proliferation after treatment with cisplatin and doxorubicin compared to MMTV-PyMT;*Apc* ^+/+^ cells. **b** Apoptosis as measured by cleaved caspase 3 immunofluorescence (IF). The percentage of apoptosis was lower in cisplatin and doxorubicin treated MMTV-PyMT;*Apc* ^Min/+^ compared to MMTV-PyMT;*Apc* ^+/+^ cells while paclitaxel treatment did not affect apoptosis levels. **c** Representative images of cleaved caspase 3 (CC3) IF. The scale bar is equal to 200 microns. *White arrows* are representative cleaved caspase 3 positive cells. Data are shown as the means ± SEM from 3 independent experiments; *P < 0.05

**Fig. 3:**
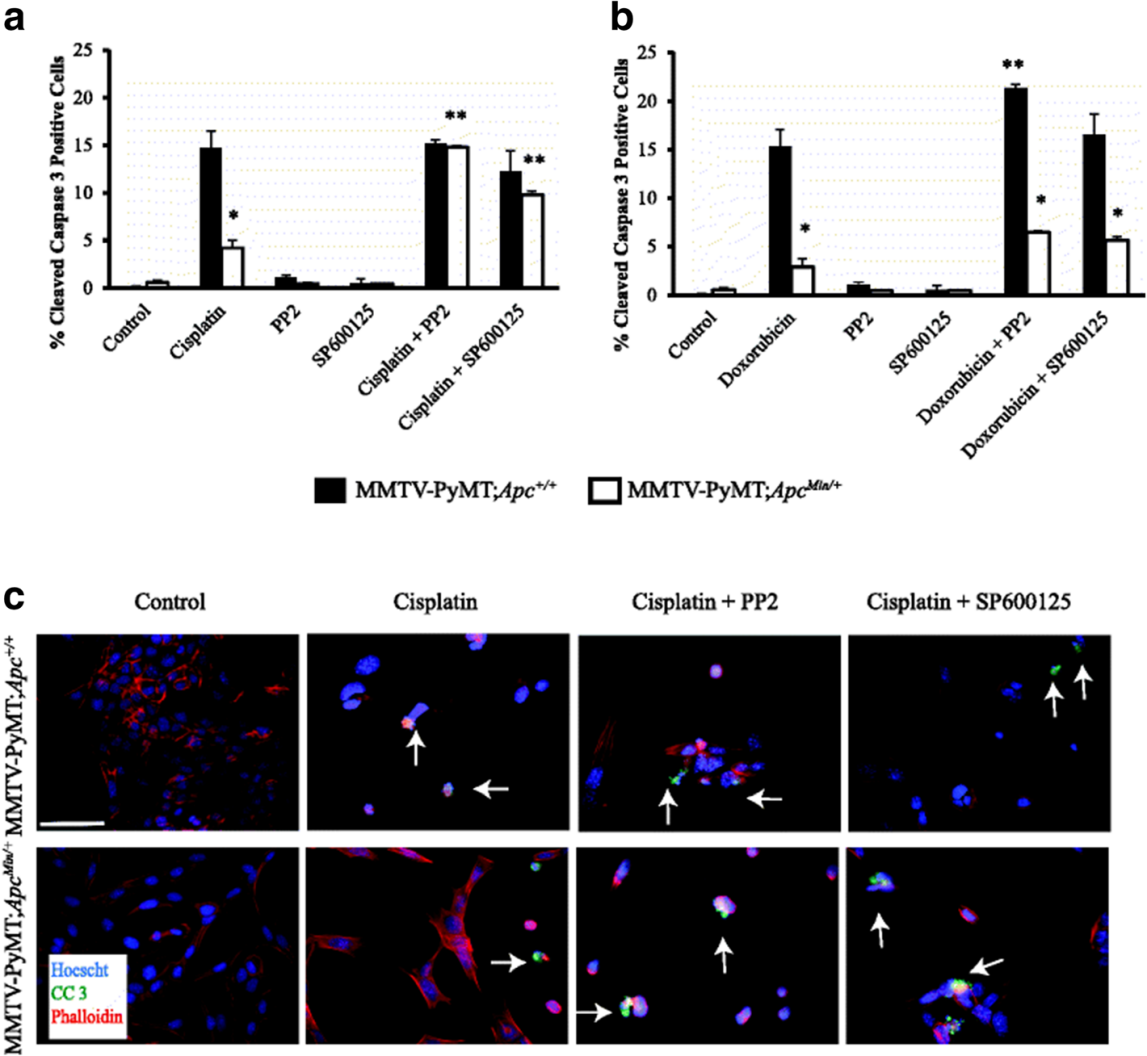
Apoptosis in MMTV-PyMT;*Apc* ^Min/+^ and MMTV-PyMT;*Apc* ^+/+^ cells treated with chemotherapeutic drugs and targeted inhibitors. **a** Apoptosis was measured by cleaved caspase 3 IF in the presence of cisplatin. Treatment with cisplatin and either PP2 or SP600125 significantly increases apoptosis compared to cisplatin alone in MMTV-PyMT;*Apc* ^Min/+^cells. No effect was observed with the addition of PP2 or SP600125 in the MMTV-PyMT;*Apc* ^+/+^cells. **b** Apoptosis was measured by cleaved caspase 3 IF with doxorubicin treatments. **c** Representative cleaved caspase 3 IF images of cells treated with cisplatin and the targeted inhibitor. The scale bar is equal to 200 microns and arrows are used to depict specific cleaved caspase 3 (CC 3) positive cells in each image. Data are shown as the means ± SEM from 3 independent experiments; *P < 0.05 when comparing MMTV-PyMT;*Apc* ^Min/+^ to MMTV-PyMT;*Apc* ^+/+^ cells and ** *P* < 0.05 when comparing the combination treatment versus a single agent (cisplatin or doxorubicin)

**Fig. 4:**
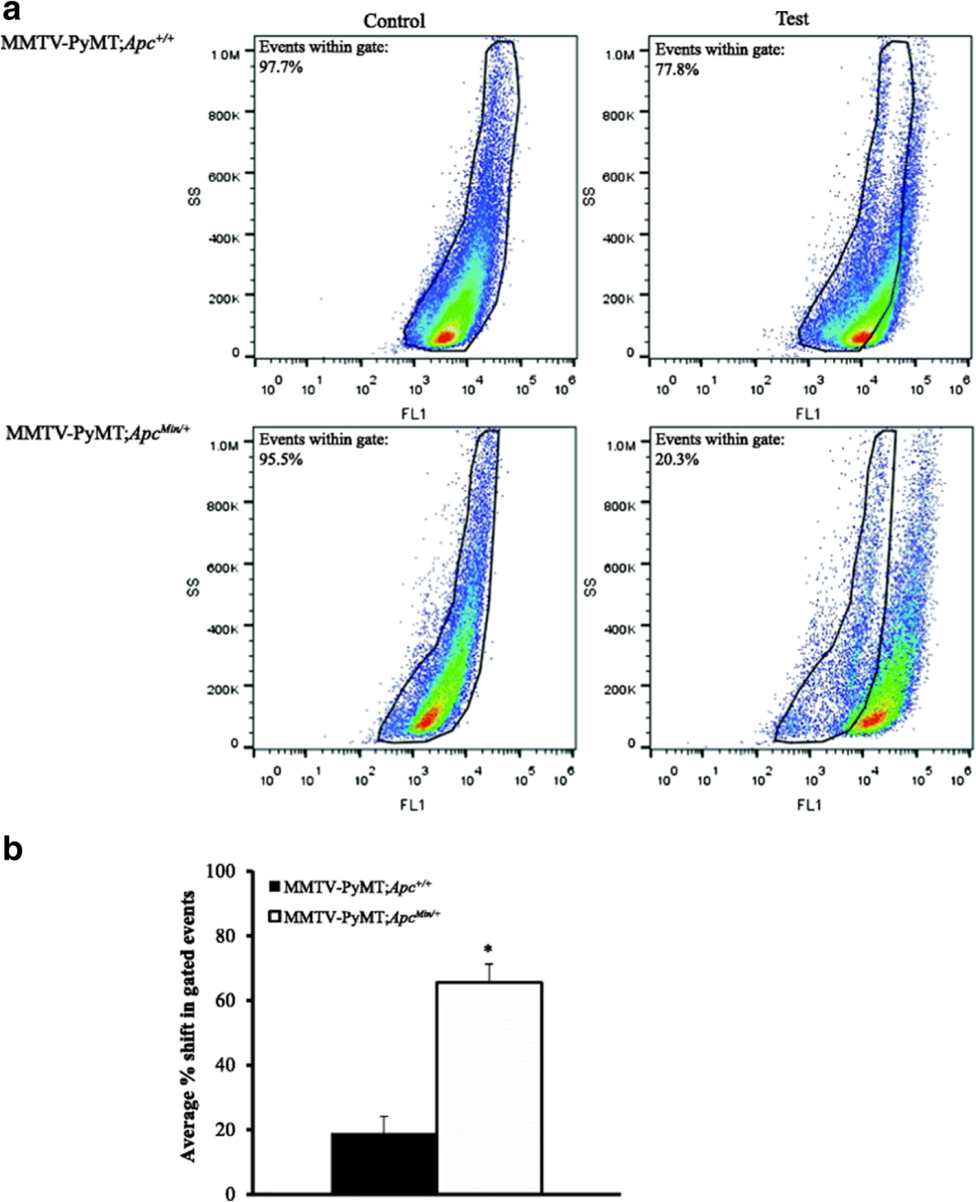
MMTV-PyMT;*Apc* ^Min/+^ cells have higher aldehyde dehydrogenase (ALDH) enzyme activity than MMTV-PyMT;*Apc* ^+/+^ cells. ALDH activity was measured using an Aldefluor™ Kit. For each cell line a Control (+DEAB) and test (−DEAB) sample were run. a Representative FACS analysis of ALDH activity in MMTVPyMT; *Apc* ^+/+^ and MMTV-PyMT;*Apc* ^Min/+^ cells using the Aldefluor™ assay. ALDH activity is increased in MMTV-PyMT;*Apc* ^Min/+^ cells. **b** The population of cells that shifted outside of the control population was calculated for each test sample, indicating ALDH activity. MMTV-PyMT;*Apc* ^Min/+^cells show a larger percentage of cells shifted outside of the control range than MMTV-PyMT;*Apc* ^+/+^cells. Data are shown as the means ± SEM from 3 independent experiments; *P < 0.05

**Fig. 5:**
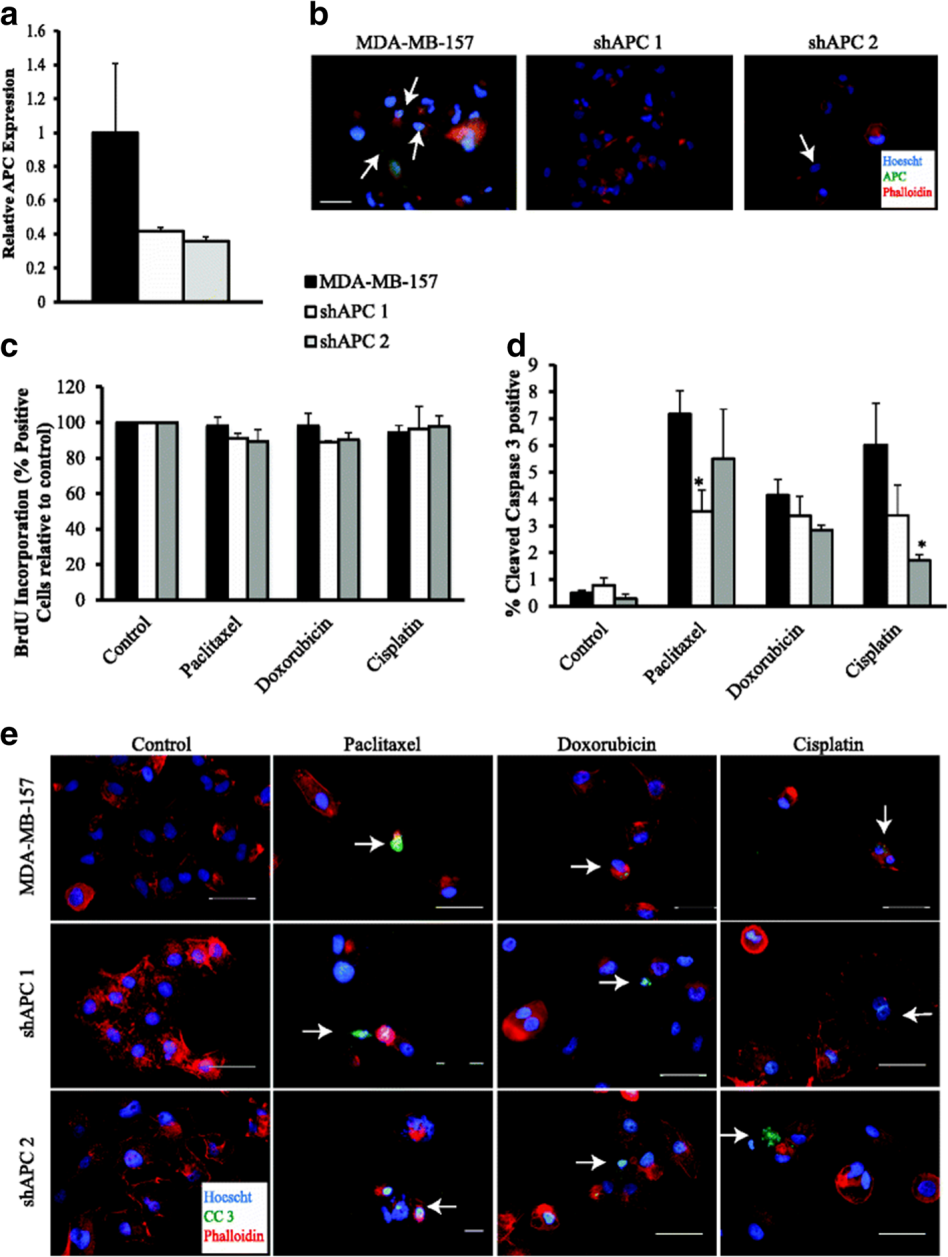
APC knockdown in MDA-MB-157 cells impacts response to paclitaxel and cisplatin. **a** Quantitative RT-PCR in MDA-MB-157 cells and shAPC constructs shows decreased level of APC in cells infected with the shAPC constructs. **b** Representative APC immunofluorescence images showing that APC knockdown cells have less APC protein compared to the MDA-MB-157 parent line. **c** Cell proliferation as measured by BrdU incorporation did not differ between the three cell lines after treatment with cisplatin, doxorubicin or paclitaxel. **d** Apoptosis as measured by cleaved caspase 3 IF. The percentage of apoptosis was lower in paclitaxel treated shAPC 1 and cisplatin treated shAPC 2 cells compared to MDA-MB-157 control cells. Doxorubicin treatment had no effect on rates of apoptosis. **e** Representative images of CC3 IF. Although there are a similar number of positive cells in many of the images, there are fewer total cells in those images representing treatments with a higher percent of apoptosis. The scale bar is equal to 100 microns (**e**) and 20 microns (**b**). Data are shown as the means ± SEM from 3 independent experiments; *P < 0.05
