# Supplementary material for: APC selectively mediates response to chemotherapeutic agents in breast cancer
Source: BMC Cancer. 2015 Jun 7;15:457. doi: 10.1186/s12885-015-1456-x (PMC4458029; doi:10.1186/s12885-015-1456-x)
Supplement: Additional file 1: — This file contains tables for each quantitative graph in the paper. Each table includes average, SEM and p-values for each cell line and treatment combination. [file 12885_2015_1456_MOESM1_ESM.docx]

Table 1.1: Multidrug resistance protein 1 gene expression in MMTV-PyMT cells corresponding to Figure 1B.

| Model | Treatment | Mean | SEM | p-value  *Apc^+/+^* vs *Apc^Min/+^* | p-value compared  to Control |
| --- | --- | --- | --- | --- | --- |
| MMTV-PyMT;*Apc^+/+^* | Control | 0.221 | 0.05 | N/A | N/A |
| MMTV-PyMT;*Apc^+/+^* | Paclitaxel | 0.633 | 0.20 | N/A | NS |
| MMTV-PyMT;*Apc^+/+^* | Cisplatin | 0.526 | 0.32 | N/A | NS |
| MMTV-PyMT;*Apc^+/+^* | Doxorubicin | 0.473 | 0.12 | N/A | NS |
| MMTV-PyMT;*Apc^Min/+^* | Control | 0.433 | 0.04 | < 0.05 | N/A |
| MMTV-PyMT;*Apc^Min/+^* | Paclitaxel | 2.263 | 0.421 | < 0.05 | < 0.05 |
| MMTV-PyMT;*Apc^Min/+^* | Cisplatin | 0.519 | 0.21 | NS | NS |
| MMTV-PyMT;*Apc^Min/+^* | Doxorubicin | 3.415 | 0.98 | < 0.05 | < 0.05 |

Table 1.2: ABCG2 gene expression in MMTV-PyMT cells corresponding to Figure 1C.

| Model | Treatment | Mean | SEM | p-value  *Apc^+/+^* vs *Apc^Min/+^* | p-value compared  to Control |
| --- | --- | --- | --- | --- | --- |
| MMTV-PyMT;*Apc^+/+^* | Control | 1.537 | 0.41 | N/A | N/A |
| MMTV-PyMT;*Apc^+/+^* | Paclitaxel | 1.433 | 0.41 | N/A | NS |
| MMTV-PyMT;*Apc^+/+^* | Cisplatin | 1.191 | 0.61 | N/A | NS |
| MMTV-PyMT;*Apc^+/+^* | Doxorubicin | 1.481 | 0.57 | N/A | NS |
| MMTV-PyMT;*Apc^Min/+^* | Control | 0.907 | 0.24 | NS | N/A |
| MMTV-PyMT;*Apc^Min/+^* | Paclitaxel | 0.893 | 0.28 | NS | NS |
| MMTV-PyMT;*Apc^Min/+^* | Cisplatin | 0.419 | 0.08 | NS | NS |
| MMTV-PyMT;*Apc^Min/+^* | Doxorubicin | 1.054 | 0.29 | NS | NS |

Table 1.3: MDR1 protein expression in MMTV-PyMT cells corresponding to Figure 1E.

| Model | Treatment | Mean | SEM | p-value  *Apc^+/+^* vs *Apc^Min/+^* | p-value compared  to Control |
| --- | --- | --- | --- | --- | --- |
| MMTV-PyMT;*Apc^+/+^* | Control | 0.783 | 0.22 | N/A | N/A |
| MMTV-PyMT;*Apc^+/+^* | Paclitaxel | 0.637 | 0.24 | N/A | NS |
| MMTV-PyMT;*Apc^+/+^* | Cisplatin | 0.746 | 0.51 | N/A | NS |
| MMTV-PyMT;*Apc^+/+^* | Doxorubicin | 0.652 | 0.03 | N/A | NS |
| MMTV-PyMT;*Apc^Min/+^* | Control | 0.943 | 0.53 | NS | N/A |
| MMTV-PyMT;*Apc^Min/+^* | Paclitaxel | 1.128 | 0.52 | NS | NS |
| MMTV-PyMT;*Apc^Min/+^* | Cisplatin | 0.417 | 0.22 | NS | NS |
| MMTV-PyMT;*Apc^Min/+^* | Doxorubicin | 1.925 | 0.16 | < 0.001 | NS |

Table 1.4: ABCG2 protein expression in MMTV-PyMT cells corresponding to Figure 1F.

| Model | Treatment | Mean | SEM | p-value  *Apc^+/+^* vs *Apc^Min/+^* | p-value compared  to Control |
| --- | --- | --- | --- | --- | --- |
| MMTV-PyMT;*Apc^+/+^* | Control | 1.569 | 0.48 | N/A | N/A |
| MMTV-PyMT;*Apc^+/+^* | Paclitaxel | 0.934 | 0.22 | N/A | NS |
| MMTV-PyMT;*Apc^+/+^* | Cisplatin | 0.870 | 0.36 | N/A | NS |
| MMTV-PyMT;*Apc^+/+^* | Doxorubicin | 1.366 | 0.56 | N/A | NS |
| MMTV-PyMT;*Apc^Min/+^* | Control | 0.009 | 0.01 | < 0.05 | N/A |
| MMTV-PyMT;*Apc^Min/+^* | Paclitaxel | 0 | 0 | <0.01 | NS |
| MMTV-PyMT;*Apc^Min/+^* | Cisplatin | 0 | 0 | NS | NS |
| MMTV-PyMT;*Apc^Min/+^* | Doxorubicin | 0 | 0 | NS | NS |

Table 1.5: BrdU Incorporation in MMTV-PyMT cells corresponding to Figure 2A

| Model | Treatment | Mean  (%) | SEM | p-value  *Apc^+/+^* vs *Apc^Min/+^* | p-value compared to Control |
| --- | --- | --- | --- | --- | --- |
| MMTV-PyMT;*Apc^+/+^* | Control | 100.00 | N/A | N/A | N/A |
| MMTV-PyMT;*Apc^+/+^* | Paclitaxel | 101.49 | 19.19 | N/A | NS |
| MMTV-PyMT;*Apc^+/+^* | Cisplatin | 52.88 | 3.83 | N/A | < 0.001 |
| MMTV-PyMT;*Apc^+/+^* | Doxorubicin | 57.97 | 6.83 | N/A | < 0.05 |
| MMTV-PyMT;*Apc^Min/+^* | Control | 100.00 | N/A | N/A | N/A |
| MMTV-PyMT;*Apc^Min/+^* | Paclitaxel | 73.56 | 22.85 | NS | NS |
| MMTV-PyMT;*Apc^Min/+^* | Cisplatin | 37.50 | 1.67 | < 0.05 | < 0.0001 |
| MMTV-PyMT;*Apc^Min/+^* | Doxorubicin | 47.50 | 2.72 | < 0.05 | < 0.0001 |

Table 1.6: Cleaved caspase 3 cell quantification in MMTV-PyMT cells corresponding to Figure 2B.

| Model | Treatment | Mean | SEM | p-value  *Apc^+/+^* vs *Apc^Min/+^* | p-value compared to Control |
| --- | --- | --- | --- | --- | --- |
| MMTV-PyMT;*Apc^+/+^* | Control | 0.362 | 0.17 | N/A | N/A |
| MMTV-PyMT;*Apc^+/+^* | Paclitaxel | 2.234 | 0.46 | N/A | < 0.01 |
| MMTV-PyMT;*Apc^+/+^* | Cisplatin | 9.553 | 1.83 | N/A | < 0.001 |
| MMTV-PyMT;*Apc^+/+^* | Doxorubicin | 21.007 | 1.41 | N/A | < 0.01 |
| MMTV-PyMT;*Apc^Min/+^* | Control | 0.024 | 0.02 | NS | N/A |
| MMTV-PyMT;*Apc^Min/+^* | Paclitaxel | 1.650 | 0.43 | NS | < 0.01 |
| MMTV-PyMT;*Apc^Min/+^* | Cisplatin | 4.18 | 0.58 | < 0.05 | < 0.05 |
| MMTV-PyMT;*Apc^Min/+^* | Doxorubicin | 10.07 | 4.23 | < 0.05 | <0.001 |

Table 1.7: Quantification of cleaved caspase 3 positive cells treated with cisplatin corresponding to Figure 3A.

| Model | Treatment | Mean | SEM | p-value  *Apc^+/+^* vs *Apc^Min/+^* | p-value compared to control | p-value to Cisplatin |
| --- | --- | --- | --- | --- | --- | --- |
| MMTV-PyMT;*Apc^+/+^* | Control | 0.119 | .05 | N/A | N/A | N/A |
| MMTV-PyMT;*Apc^+/+^* | Cisplatin | 14.751 | 1.75 | N/A | < 0.001 | N/A |
| MMTV-PyMT;*Apc^+/+^* | PP2 | 1.104 | .26 | N/A | NS | N/A |
| MMTV-PyMT;*Apc^+/+^* | SP600125 | 0.545 | .45 | N/A | NS | N/A |
| MMTV-PyMT;*Apc^+/+^* | Cisplatin + PP2 | 15.185 | 2.12 | N/A | < 0.001 | NS |
| MMTV-PyMT;*Apc^+/+^* | Cisplatin + SP600125 | 12.277 | 1.41 | N/A | < 0.001 | NS |
| MMTV-PyMT;*Apc^Min/+^* | Control | 0.587 | .26 | NS | N/A | N/A |
| MMTV-PyMT;*Apc^Min/+^* | Cisplatin | 4.180 | .83 | < 0.001 | < 0.05 | N/A |
| MMTV-PyMT;*Apc^Min/+^* | PP2 | 0.480 | .07 | NS | NS | N/A |
| MMTV-PyMT;*Apc^Min/+^* | SP600125 | 0.476 | .07 | NS | NS | N/A |
| MMTV-PyMT;*Apc^Min/+^* | Cisplatin + PP2 | 14.801 | .36 | NS | < 0.001 | < 0.001 |
| MMTV-PyMT;*Apc^Min/+^* | Cisplatin + SP600125 | 9.811 | 2.27 | NS | < 0.001 | < 0.001 |

Table 1.8: Quantification of cleaved caspase 3 positive cells treated with doxorubicin corresponding to Figure 3B.

| Model | Treatment | Mean | SEM | p-value  *Apc^+/+^* vs *Apc^Min/+^* | p-value compared to control | p-value to Doxorubicin | |
| --- | --- | --- | --- | --- | --- | --- | --- |
| MMTV-PyMT;*Apc^+/+^* | Control | 0.119 | 0.05 | N/A | N/A | | N/A |
| MMTV-PyMT;*Apc^+/+^* | Doxorubicin | 15.363 | 0.92 | N/A | < 0.001 | | N/A |
| MMTV-PyMT;*Apc^+/+^* | PP2 | 1.104 | 0.26 | N/A | NS | | N/A |
| MMTV-PyMT;*Apc^+/+^* | SP600125 | 0.545 | 0.45 | N/A | NS | | N/A |
| MMTV-PyMT;*Apc^+/+^* | Doxorubicin + PP2 | 21.33 | 0.60 | N/A | < 0.001 | | NS |
| MMTV-PyMT;*Apc^+/+^* | Doxorubicin + SP600125 | 16.54 | 3.59 | N/A | < 0.001 | | NS |
| MMTV-PyMT;*Apc^Min/+^* | Control | 0.587 | 0.26 | NS | N/A | | N/A |
| MMTV-PyMT;*Apc^Min/+^* | Doxorubicin | 2.91 | 0.46 | < 0.001 | < 0.05 | | N/A |
| MMTV-PyMT;*Apc^Min/+^* | PP2 | 0.480 | 0.07 | NS | NS | | N/A |
| MMTV-PyMT;*Apc^Min/+^* | SP600125 | 0.476 | 0.07 | NS | NS | | N/A |
| MMTV-PyMT;*Apc^Min/+^* | Doxorubicin + PP2 | 6.44 | 1.98 | < 0.001 | < 0.001 | | < 0.001 |
| MMTV-PyMT;*Apc^Min/+^* | Doxorubicin + SP600125 | 5.66 | 2.78 | < 0.001 | < 0.001 | | < 0.001 |

Table 1.9: BrdU Incorporation values in MDA-MB-157 and shAPC cells corresponding to Figure 5B

| Model | Treatment | | Mean  (%) | SEM | p-value compared  to MDA-MB-157 | p-value compared  to control | |
| --- | --- | --- | --- | --- | --- | --- | --- |
| MDA-MB-157 | | Control | 100 | N/A | N/A | N/A |  |
| MDA-MB-157 | | Paclitaxel | 98.02 | 5.00 | N/A | NS |  |
| MDA-MB-157 | | Cisplatin | 94.38 | 3.76 | N/A | NS |  |
| MDA-MB-157 | | Doxorubicin | 98.10 | 7.00 | N/A | NS |  |
| shAPC1 | | Control | 100 | N/A | NS | N/A |  |
| shAPC1 | | Paclitaxel | 91.12 | 2.67 | NS | NS |  |
| shAPC1 | | Cisplatin | 96.38 | 12.72 | NS | NS |  |
| shAPC1 | | Doxorubicin | 88.97 | 0.55 | NS | NS |  |
| shAPC2 | | Control | 100 | N/A | NS | N/A |  |
| shAPC2 | | Paclitaxel | 77.62 | 6.62 | NS | NS |  |
| shAPC2 | | Cisplatin | 93.89 | 5.98 | NS | NS |  |
| shAPC2 | | Doxorubicin | 89.51 | 3.79 | NS | NS |  |

Table 1.10: Quantification of cleaved caspase 3 positive cells in MDA-MB-157 and shAPC cells corresponding to Figure 5C.

| Model | Treatment | | Mean  (%) | SEM | p-value compared  to MDA-MB-157 | p-value compared  to control | |
| --- | --- | --- | --- | --- | --- | --- | --- |
| MDA-MB-157 | | Control | 0.506 | 0.08 | N/A | N/A |  |
| MDA-MB-157 | | Paclitaxel | 7.178 | 0.87 | N/A | < 0.01 |  |
| MDA-MB-157 | | Cisplatin | 6.019 | 1.55 | N/A | < 0.01 |  |
| MDA-MB-157 | | Doxorubicin | 4.158 | 0.57 | N/A | < 0.05 |  |
| shAPC1 | | Control | 0.789 | 0.78 | NS | N/A |  |
| shAPC1 | | Paclitaxel | 3.543 | 0.79 | < 0.05 | < 0.05 |  |
| shAPC1 | | Cisplatin | 3.394 | 1.13 | NS | < 0.05 |  |
| shAPC1 | | Doxorubicin | 3.387 | 0.72 | NS | NS |  |
| shAPC2 | | Control | 0.283 | 0.15 | NS | N/A |  |
| shAPC2 | | Paclitaxel | 5.512 | 1.84 | NS | NS |  |
| shAPC2 | | Cisplatin | 1.722 | 0.20 | NS | < 0.01 |  |
| shAPC2 | | Doxorubicin | 2.805 | 0.18 | < 0.05 | < 0.05 |  |
